# Added diagnostic value of routinely measured hematology variables in diagnosing immune checkpoint inhibitor mediated toxicity in the emergency department

**DOI:** 10.1002/cam4.5956

**Published:** 2023-04-19

**Authors:** Michael S. A. Niemantsverdriet, Bram E. L. Vrijsen, Therese Visser ’t Hooft, Karijn P. M. Suijkerbuijk, Wouter W. van Solinge, Maarten J. ten Berg, Saskia Haitjema

**Affiliations:** ^1^ Central Diagnostic Laboratory University Medical Center Utrecht, Utrecht University Utrecht The Netherlands; ^2^ SkylineDx Rotterdam The Netherlands; ^3^ Department of Internal Medicine University Medical Center Utrecht, Utrecht University Utrecht The Netherlands; ^4^ Department of Medical Oncology University Medical Center Utrecht, Utrecht University Utrecht The Netherlands

**Keywords:** biomarkers, emergency department, immune checkpoint inhibitor, immune‐related adverse events, machine learning

## Abstract

**Background:**

Immune checkpoint inhibitors (ICI) show remarkable results in cancer treatment, but at the cost of immune‐related adverse events (irAE). irAE can be difficult to differentiate from infections or tumor progression, thereby challenging treatment, especially in the emergency department (ED) where time and clinical information are limited. As infections are traceable in blood, we were interested in the added diagnostic value of routinely measured hematological blood cell characteristics in addition to standard diagnostic practice in the ED to aid irAE assessment.

**Methods:**

Hematological variables routinely measured with our hematological analyzer (*Abbott CELL‐DYN Sapphire*) were retrieved from Utrecht Patient Oriented Database (UPOD) for all patients treated with ICI who visited the ED between 2013 and 2020. To assess the added diagnostic value, we developed and compared two models; a base logistic regression model trained on the preliminary diagnosis at the ED, sex, and gender, and an extended model trained with lasso that also assessed the hematology variables.

**Results:**

A total of 413 ED visits were used in this analysis. The extended model showed an improvement in performance (area under the receiver operator characteristic curve) over the base model, 0.79 (95% CI 0.75–0.84), and 0.67 (95% CI 0.60–0.73), respectively. Two standard blood count variables (eosinophil granulocyte count and red blood cell count) and two advanced variables (coefficient of variance of neutrophil depolarization and red blood cell distribution width) were associated with irAE.

**Conclusion:**

Hematological variables are a valuable and inexpensive aid for irAE diagnosis in the ED. Further exploration of the predictive hematological variables could yield new insights into the pathophysiology underlying irAE and in distinguishing irAE from other inflammatory conditions.

## Graphical abstract

Routinely measured hematological parameters have added diagnostic value in patients on checkpoint inhibitors who visit the emergency department with suspected immune‐related adverse events. The physician's preliminary diagnosis had an AUROC of 0.67, which improved to 0.79 after adding four hematological parameters.

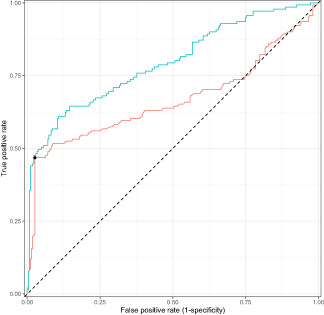

## 1 INTRODUCTION

Within the immunotherapeutic field of cancer treatment, multiple new and promising treatment options have emerged over the past years.^1^ Among these, immune checkpoint inhibitors (ICI) are increasingly being used as an oncologic treatment strategy for multiple types of cancer and have drastically improved survival of responding patients. For example, patients with advanced melanoma treated with combined nivolumab and ipilimumab therapy have shown to result in a median overall survival of over 60 months,^2^ whereas the median survival of patients with metastatic melanoma used to be less than 1 year before the introduction of checkpoint inhibitors.^3^ The proportion of cancer patients benefiting from ICI is increasing rapidly, with now over 40% of cancer patients qualifying for ICI treatment.^4^ However, their use is associated with a wide variety of immune‐related adverse events (irAE), such as auto‐immune colitis and pneumonitis.^5^ Because of overlap in clinical presentation, it can be difficult to differentiate these irAE from progressive disease or other inflammatory conditions, such as infections. Especially in the emergency department (ED) where time and resources are limited, this may lead to diagnostic delay, inappropriate treatment, and a considerable amount of (unnecessary) diagnostic testing.^6^, ^7^ Accurate and early diagnosis of patients presenting in the ED with irAE is therefore key to start adequate treatment as soon as possible.^8^, ^9^

Currently, there are only a few biomarkers available that can aid in diagnosing irAE.^6^, ^10^ A solution to this problem might be found in routinely measured hematological variables. Bacterial infection and viral infections are commonly characterized by high neutrophil and lymphocyte counts respectively, whereas auto‐immune diseases and allergies typically show high eosinophil counts. Previous research has found associations between irAE and increased counts of standard hematology measurements (e.g., absolute lymphocyte count and eosinophil count).^6^ In addition, changes in B‐ and T‐cell receptor repertoire show associations with irAE onset and prognosis.^6^ However, none of these biomarkers have been extensively validated or are used in clinical practice. Most modern hematology analyzers not only provide blood cell counts, but also measure morphologic characteristics, such as cell size, intrinsic properties and cell viability that carry diagnostic and prognostic value. This raises the question whether they may also be of use in the setting of immunological toxicity.^11^, ^12^, ^13^

To answer these types of questions, scrutinizing complex datasets with conventional statistical methods, such as logistic regression, do not provide stable estimates of the variable's coefficients as models contain too many variables and a low number of samples. New advanced statistical and machine learning (ML) methods are able to remove irrelevant variables thereby reducing the number of variables. In addition, variables of high importance, also known as predictors, can be identified by evaluating the trained coefficient of the trained model. This way, ML allows for the possible identification of new biomarkers and exploration of new horizons in research to aid irAE diagnosis.

The aim of this study was therefore to determine the added value of routinely measured hematology characteristics, modeled through ML, as compared to the standard diagnostic practice. This may aid in the diagnosis of irAE in the ED and understanding of the pathophysiology.

## 2 METHODS

### 2.1 Study population

This retrospective observational study included all visits to the ED of the University Medical Center Utrecht (UMC Utrecht) between 2013 and 2020 of patients who were being treated with any type of ICI for any type of cancer, until 3 months after cessation of treatment. Because irAE can occur even after cessation of treatment, we chose to include ED visits up to 3 months after treatment with ICI ended.^14^ The cutoff of 3 months was chosen after discussion between the authors. If patients had more than one disease episode (defined as a consecutive period with infection‐like symptoms), all patient's ED visits were included separately, whereas for patients with multiple ED visits during one disease episode, only the first visit was included. If patients visited the ED multiple times for the same condition (e.g., due to worsening of symptoms), only the first visit was included.

### 2.2 Data collection

For all ED visits, demographic (age and sex), medication, and hematology data were extracted from the Utrecht Patient Orientated Database (UPOD). In brief, UPOD is a relational database combining clinical characteristics, medication, and laboratory measurements of patients in the UMC Utrecht since 2004.^15^ We used hematological variables measured by the *CELL‐DYN Sapphire* hematology analyzer (*Abbott diagnostics*). The *CELL‐DYN Sapphire* is a cell counter equipped with a 488‐nm blue diode laser and uses multiple techniques, such as electrical impedance, spectrophotometry, and laser light scattering, to measure morphological characteristics of leukocytes (incl. 5‐part differential), red blood cells (RBCs), and platelets for both classification and enumeration. Each time a component of a complete blood cell count (CBC) is requested, all data generated by the hematology analyzer are automatically stored in UPOD, including a substantial number of raw and research‐only values and background data on cell characteristics which are made available for research purposes. Only visits with available Sapphire data within the first 4 h after ED presentation were included in this study to ensure we only used data from patients with infection‐like symptoms during the ED visit. UPOD data acquisition and management is in accordance with current regulations concerning privacy and ethics.

### 2.3 irAE label definition

A manual chart review was done for all ED visits within our study population by two of the authors (TVtH and BV). Visits for evidently unrelated conditions were excluded. We recorded both the preliminary and definite diagnosis. The preliminary diagnosis was defined as the diagnosis made by the treating physician in the ED and was characterized as either *suspected irAE* or *other*. The definitive diagnosis was defined as the diagnosis made by the treating physician at discharge from the hospital or at the end of treatment and was characterized as *irAE* or *other*. Ambiguous cases were resolved through consensus.

### 2.4 Model development

Two models were trained to evaluate the added diagnostic performance of the hematology variables for irAE diagnosis. The first model (base) assessed the preliminary diagnosis, sex, and age with logistic regression thereby imitating clinical practice at the ED, whereas the second model (extended) also included the 77 additional hematology variables. A quality control protocol was performed to remove variables with no additional predictive value during model development: hematology variables with a Pearson correlation of >0.80 or with low number of unique (*n* = 5) values were removed. The extended model was trained using lasso machine learning that can automatically reduce the number of variables, thereby reducing the risk of overfitting and aiding the interpretability of the model. Means and standard deviations are shown for normally distributed variables whereas medians and inter‐quartile ranges (IQR) are shown for non‐normally distributed variables.

Model performance was assessed using cross validation (CV). With CV, the data are split in *K* number of partitions (folds), of which *K‐1* folds are used for training and *1* for testing. This exercise is repeated *K* times resulting in *K* models with *K* performance estimates. Contrary to the conventional train‐and‐test split, multiple models are trained on multiple data splits, thereby using all data to assess the model's performance. The lasso algorithm performs shrinkage of coefficients that can get as small as 0, thereby removing variables. The lambda hyper‐parameter of lasso determines the degree of shrinkage and was optimized in a double loop cross‐validation (DLCV) scheme, also known as nested cross validation (Figure S1).^16^ A *K* of 10 was used for both the CV and DLCV schemes.

### 2.5 Model evaluation

The discrimination of models was assessed by plotting receiver operator characteristic (ROC) curves. The area under the ROC (AUROC) is a measure of discrimination, an AUROC of 1 indicates a perfect model, whereas an AUROC 0.5 indicates a random model. The 95% confidence interval (CI) of the AUROC was computed with the R cvAUC package by evaluating the test performances of the two model configurations trained in both CV schemes.^17^ Variable coefficients of the ten models trained in the DLCV were evaluated as variable importance (predictors).

The clinical application and value of the trained models was evaluated with both calibration plots and net benefit curves. Calibration plots portray the agreement between predicted probabilities and the observed frequency of irAE. A calibration with an intercept 0 and slope of 1 shows perfect calibration, whereas a slope of >1 shows a model that overestimates outcome and a slope of <1 underestimates diagnosis. 80% and 95% CI intervals of the calibration plots were generated with the R givitR package.^18^ Net benefit is a measure to evaluate the clinical benefit of a prediction model by comparing the benefit [treating diseased, true positives (TP)] and cost [treating non‐diseased, false‐positive (FP)].^19^ Net benefit is assessed by subtracting the cost from the benefit for the complete range of predictions values ($p_{t}$). Formula 1 shows that the net benefit increases by the number of TP and is penalized by the number of non‐diseased (FP), especially when the prediction threshold value increases $\left(\frac{p_{t}}{1-p_{t}}\right)$. Besides the net benefit, the number needed to treat (NNT) is shown as a comparison to how healthcare professionals consider whether the patient has a specific illness or that treatment is required. All analyses were performed in R version 4.1.2.^20^

(1) $$\mathrm{net}\;\text{benefit}\;\left(p_{t}\right)=\frac{\mathrm{TP}}{n}-\frac{\mathrm{FP}}{n}\left(\frac{p_{t}}{1-p_{t}}\right)$$

### 2.6 Post hoc subgroup analysis

To assess the independence of the identified biomarkers we adjusted for the baseline clinical variables, we performed a multivariate analysis including the identified biomarkers, age, sex, cancer type, and ICI medication. To reduce the number of coefficients and to remove groups with low prevalence, various cancer types, and ICI medications were grouped.

A second post hoc analysis was performed to check whether the identified biomarkers were associated with disease severity as measured by CTCAE grade.

## 3 RESULTS

### 3.1 Patient characteristics

Between 2013 and 2020, 409 ED visits of 257 patients who were treated with ICI and had available blood counts were included in this study (mean ED visits per patient 1.6). The irAE diagnosis of 91 visits were inconclusive from the medical records, of which the diagnosis was later adjusted in 24 cases. In both the *other* (*n* = 268) and *irAE* (*n* = 141) sub‐groups there were more males, 63.1% and 64.5%, respectively (Table 1). Mean age did not differ between the *other* (62.2) and *irAE* group (61.7). The use of both ipilimumab and nivolumab were significantly higher in the *irAE* group (*p* < 0.01), whereas the use of nivolumab and pembrolizumab were significantly lower in the *irAE* group (*p* < 0.01). An overview of the irAE diagnoses is shown in Table S1.

**TABLE 1:** Cohort baseline characteristics of emergency department visits treated with immune checkpoint inhibitors.

	Other (*n* = 268)	irAE (*n* = 141)	*p*‐Values
Age (SD)	62.2 (11.9)	61.7 (12.8)	0.68
Sex, male count (%)	169 (63.1%)	91 (64.5%)	0.851
*Cancer diagnosis*			
Central nervous system	4 (1.5%)	3 (2.1%)	
Gynecological	6 (2.2%)	2 (1.4%)	
Head and neck	3 (1.1%)	2 (1.4%)	
Hematological	7 (2.6%)	4 (2.8%)	
Hepta‐pancreato‐biliary	2 (0.7%)	1 (0.7%)	
Intestinal	14 (5.2%)	0 (0.0%)	
Lung	88 (32.8%)	23 (16.3%)	
Skin	119 (44.4%)	94 (66.7%)	
Urological	25 (9.3%)	12 (8.5%)	
*Preliminary diagnosis, count*			
Other	261	75	<0.001
irAE	7	66	
*CTCAE* ^b^ *grade*			
1		13 (9.2%)	
2		48 (34.0%)	
3		69 (48.9%)	
4		10 (7.1%)	
5		1 (0.7%)	
*ICI medication* ^a^, count (%)			
Atezolizumab	16 (6.0%)	2 (1.4%)	0.06
Durvalumab	4 (1.5%)	5 (3.5%)	0.322
Ipilimumab	25 (9.3%)	26 (18.4%)	0.013
Nivolumab	89 (33.2%)	24 (17.0%)	0.001
Pembrolizumab	86 (32.1%)	26 (18.4%)	0.005
Tremelimumab	5 (1.9%)	4 (2.8%)	0.778
Ipilimumab and nivolumab	43 (16.0%)	54 (38.3%)	<0.001

Abbreviations: ICI, immune checkpoint inhibitor; irAE, immune‐related adverse events.

^a^ Not mutually exclusive.

^b^ Common terminology criteria adverse events.

### 3.2 Model performance

**FIGURE 1:**
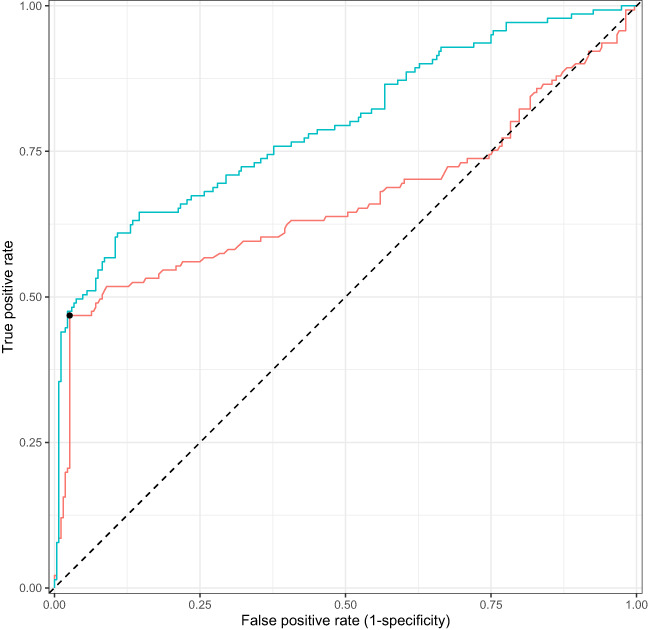
ROC of the base (red line) and extended (blue line) models test predictions. Predictions on the test folds of the double loop cross validation scheme were concatenated to draw the ROC curves. The black dot denotes the discriminative metrics of the preliminary diagnosis. The diagonal line shows the performance of a random model.

After removing variables that did not meet our quality control criteria, 53 of the 77 Sapphire variables were used in the extended model (Table S2 and Figure S2). The base model had an AUROC of 0.67 (0.60–0.79 95% CI) and the extended model had an AUROC of 0.79 (0.75–0.84 95% CI), a difference in 0.13. The training performance was marginally higher for both the base and extended model as compared to the test performance, 0.74 (0.72–0.76 95% CI) and 0.86 (0.84–0.87 95% CI), respectively, providing evidence there was no overfitting. In line with the AUROC metrics, the extended model trained on all data shows the best ROC and PRC curves (Figure 1).

### 3.3 Discriminative metrics

**FIGURE 2:**
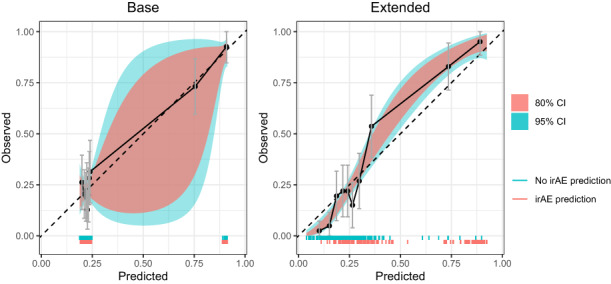
Calibration plots of both the base and extended models. Both calibration curves computed with the number of expected (model predictions) and observed irAE are shown, as well as the 80% and 90% confidence intervals (CI). The segments on the lower part of both plots indicate the computed predictions for each model.

**FIGURE 3:**
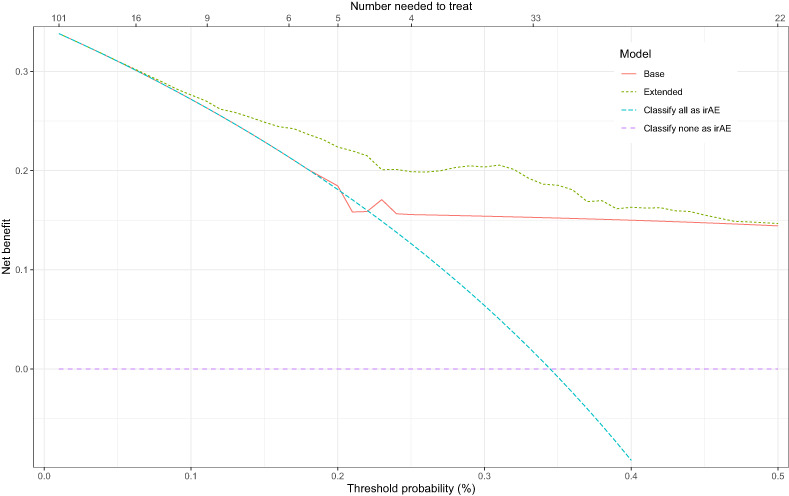
Decision curve of the base and extended models. The blue and purple indicate the extreme base strategies of either treating everyone as irAE or none as irAE, respectively.

To assess the potential value in clinical practice of the extended model, predictions of the base and extended models were evaluated with both calibration and net benefit plots. The extended model showed better calibration than the base model (Figure 2). The 95% CI of the base model are very wide compared to the extended model and the predictions of the extended model are more equally distributed. In addition, decision curve analysis showed improved net benefit of the extended model as compared to the base model over the complete threshold probability range (Figure 3).

### 3.4 Variable importance

**FIGURE 4:**
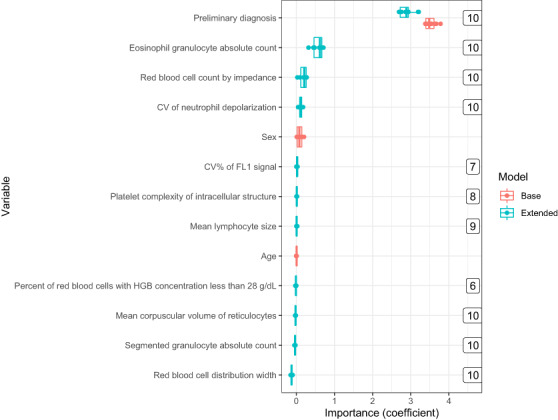
Variable importance of both the base and extended model. Only variables selected more than five times by lasso in the DLCV are shown. The number positioned on right shows the number of times a variable was selected by the extended model in the 10‐fold DLCV scheme.

Variables' coefficients, as well as the number of times a variable was selected by the extended model, were documented during training, and are shown in Figure 4 and Table 2. The preliminary diagnosis was highly predictive for irAE diagnosis in both the base and extended model with a coefficient of 3.53 ± 0.14 and 2.88 ± 0.18, respectively. The extended model also identified the following Sapphire variables as predictors for irAE diagnosis: number of eosinophils (eos), red blood cell count measured with impedance (rbci), coefficient of variance neutrophil depolarization (ndcv), and red blood cell distribution width (rdw), of which the latter was negatively associated with irAE. Eos was highly correlated with percentage of eosinophils (peos) and rbci with other red blood cell measurements variables (rbco, hgb, and hct) (Table S2). The sex and age variables were not selected by lasso in any of the ten iterations in the DLCV scheme.

**TABLE 2:** Variable importance for both the base and extended models estimated in the CV schemes.

Definition	Estimated coefficient	
	Base	Extended
Preliminary diagnosis	3.52 (±0.14)	2.89 (±0.19)
Eosinophil granulocyte absolute count		0.57 (±0.13)
Red blood cell count by impedance		0.18 (±0.10)
Coefficient of variance of neutrophil side scattering		0.11 (±0.04)
Red blood cell distribution width		−0.12 (±0.02)

*Note*: Top 5 selected variables based on absolute coefficient mean (±SD) for both models.

### 3.5 Post hoc subgroup analysis

After adjusting for age, sex, cancer type (grouped as skin, lung, urological or other) and ICI medication (grouped as ipilimumab, nivolumab, pembrolizumab, ipilimumab, and nivolumab, or other) we found that three of the four identified variables were still significantly associated with irAE, namely: eos (*p*‐value 0.0144), rbci (*p*‐value 0.0035), and rdw (*p*‐value 0.0003). In this model we did not find a significant association for ndcv (*p*‐value 0.0781). Furthermore, we did not find an association between the values of the identified variables and the irAE severity as measured by CTCAE grade (Supplementary Figure).

## 4 DISCUSSION

Accurate identification of irAE in patients using ICI in the ED is of vital importance to guide treatment decisions. With new statistical methods and ML, we explored the possible added diagnostic value of 77 hematological variables measured by the *CELL‐DYN Sapphire* in diagnosing irAE in patients using ICI as compared to standard clinical practice. The extended model showed improvement in discrimination, calibration, and net benefit as compared to the base model, indicating that the hematological variables indeed have added value in the diagnostic process of identifying irAE in patients using ICI in the emergency department setting.

Our extended model showed better performance as well as calibration over the base model. However, due to the low number of values of the base model and the good predictive performance of the preliminary diagnosis, the predictions of the base model were not equally distributed. The net benefit of the extended model was better than the base model, especially in the therapeutic range around 25%. The exact threshold for the number needed to treat will vary depending on the characteristics of the individual patient and the severity of the symptoms. A false‐positive diagnosis of irAE will lead to cessation of the checkpoint inhibitor, which would possibly withhold a life‐saving therapy from the patient. On the other hand, a false‐negative diagnosis will lead to a delayed treatment for irAE, which is potentially fatal.^21^

Of all variables, the preliminary diagnosis was deemed highly important by both the base and extended models indicating that the first diagnosis of the physician is a very good proxy for irAE diagnosis. Both age and sex showed low importance in the base model and were not selected by the lasso algorithm in any of the 10 DLCV iterations, which is in line with existing evidence.^22^ Interestingly, only a few of the 77 hematological variables were selected by the lasso algorithm in each iteration. This diagnostic study cannot not determine causality. However, a causal relationship can be postulated based on the literature.

Eosinophiles are thought to play a pathogenic role in auto‐immune disorders and are known to be associated with irAE.^6^ Neutrophil depolarization is a feature of neutrophil activation, which has also been associated with auto‐immunity, but this has not been studied extensively.^23^ We found the red blood cell distribution width (rdw) to be negatively associated with irAE. Increased rdw is known to be associated with infections, which are arguably the most likely alternative diagnosis when considering irAE.^24^

Our study has some limitations. The population is highly heterogeneous, with multiple types of tumors and treatments. This may have hampered the identification of a specific predictor for a particular subset of patients. Unfortunately, we did not have enough data to stratify patients based on either cancer type or medication. Even though the post hoc group analysis showed significant results for 3 of the 4 identified variables after adjusting for the baseline characteristics, future research is needed to validate these results. Moreover, the diagnoses were retrospectively defined or changed as our data was collected on routine basis.

To our knowledge, this study is one of the first of its kind in exploring the diagnostic potential of these raw and research‐only hematological variables using ML in the emergency department setting. Since the raw data from this type of hematology analyzer are not ubiquitously available, we were not able to externally validate our results. As a result, this study has to be viewed as exploratory and more research is required before these hematological variables, either individually or in a model, can be used in clinical practice. The diagnostic performance of such a model might be improved by combining hematological variables with other new sets of biomarkers, as well as the preliminary diagnosis.

This study raises the question if the hematological variables might also have diagnostic value in the setting of other diseases and treatments.^11^, ^12^, ^13^ As they are inexpensive and relatively easily and rapidly obtained in general blood counts, they could be an interesting new tool in future diagnostic research. As shown here, a clinical diagnostic model may aid the clinical decision‐making process of a physician by providing a continuous prediction score that can be combined with the professional interpretation by a clinical chemist to accommodate integral diagnostics of a patient's clinical state.^25^ Instead of looking at differences between patients using cross‐sectional data, within‐patient differences may be a better approximation of a patient's health trajectory potentially allowing for predicting the incidence of irAE at the start of ICI treatment.

Overall, we show that hematological variables show diagnostic performance in the identification of irAE in patients using ICI at the ED and that they have added value compared to standard diagnostic practice. Our results suggest new directions for further research using (advanced) hematological variables for irAE diagnosis in the emergency setting.

## AUTHOR CONTRIBUTIONS

**Michael S. A. Niemantsverdriet:** Conceptualization (equal); formal analysis (equal); methodology (equal); writing – original draft (equal). **Bram E. L. Vrijsen:** Conceptualization (equal); data curation (equal); formal analysis (equal); writing – original draft (equal). **Thérèse Visser 't Hooft:** Data curation (equal). **Karijn P. M. Suijkerbuijk:** Data curation (equal). **Wouter W. van Solinge:** Supervision (equal). **Maarten J. ten Berg:** Conceptualization (equal); formal analysis (equal). **Saskia Haitjema:** Conceptualization (equal); data curation (equal); formal analysis (equal).

## FUNDING INFORMATION

None.

## CONFLICT OF INTEREST STATEMENT

MN is employed by SkylineDx, Rotterdam and receives a PhD fellowship from SkylineDx, Rotterdam. KS: Consulting/advisory relationship: Bristol Myers Squibb, Merck Sharp and Dome, Abbvie, Pierre Fabre, Novartis. Honoraria received: Novartis, Roche, Merck Sharp and Dome. Research funding, TigaTx, Bristol Myers Squibb, Philips, unrelated to this project. All paid to institution and outside the submitted work. The remaining authors declare that the research was conducted in the absence of any commercial or financial relationships that could be construed as a potential conflict of interest.

## ETHICS STATEMENT

This study was performed in accordance with the Declaration of Helsinki and the ethical guidelines of our institution. The institutional review board of the UMC Utrecht approved this study (reference number 20–591/C) and waived the need for informed consent as only pseudonymized data were used for this study. Data collection and handling was conducted in accordance with European privacy legislation (GDPR).

## Supporting information


Data S1:

## Data Availability

Data from the UMC are available upon reasonable request from the corresponding author (S. Haitjema).

## References

[1] Farkona S , Diamandis EP , Blasutig IM . Cancer immunotherapy: the beginning of the end of cancer? BMC Med. 2016;14(1):73. 27151159 10.1186/s12916-016-0623-5 PMC4858828

[2] Larkin J , Chiarion‐Sileni V , Gonzalez R , et al. Five‐year survival with combined nivolumab and ipilimumab in advanced melanoma. N Engl J Med. 2019;381(16):1535‐1546. 31562797 10.1056/NEJMoa1910836

[3] Robert C , Long GV , Brady B , et al. Nivolumab in previously untreated melanoma without BRAF mutation. N Engl J Med. 2015;372(4):320‐330. 25399552 10.1056/NEJMoa1412082

[4] Haslam A , Prasad V . Estimation of the percentage of US patients with cancer who are eligible for and respond to checkpoint inhibitor immunotherapy drugs. JAMA Netw Open. 2019;2(5):e192535‐e. 31050774 10.1001/jamanetworkopen.2019.2535 PMC6503493

[5] Martins F , Sofiya L , Sykiotis GP , et al. Adverse effects of immune‐checkpoint inhibitors: epidemiology, management and surveillance. Nat Rev Clin Oncol. 2019;16(9):563‐580. 31092901 10.1038/s41571-019-0218-0

[6] Hommes JW , Verheijden RJ , Suijkerbuijk KPM , Hamann D . Biomarkers of checkpoint inhibitor induced immune‐related adverse events‐a comprehensive review. Front Oncol. 2020;10:585311. 33643899 10.3389/fonc.2020.585311 PMC7905347

[7] von Itzstein MS , Khan S , Gerber DE . Investigational biomarkers for checkpoint inhibitor immune‐related adverse event prediction and diagnosis. Clin Chem. 2020;66(6):779‐793. 32363387 10.1093/clinchem/hvaa081 PMC7259479

[8] Puzanov I , Diab A , Abdallah K , et al. Managing toxicities associated with immune checkpoint inhibitors: consensus recommendations from the Society for Immunotherapy of cancer (SITC) toxicity management working group. J Immunother Cancer. 2017;5(1):95. 29162153 10.1186/s40425-017-0300-z PMC5697162

[9] La‐Beck NM , Jean GW , Huynh C , Alzghari SK , Lowe DB . Immune checkpoint inhibitors: new insights and current place in cancer therapy. Pharmacotherapy. 2015;35(10):963‐976. 26497482 10.1002/phar.1643

[10] Jia XH , Geng LY , Jiang PP , et al. The biomarkers related to immune related adverse events caused by immune checkpoint inhibitors. J Exp Clin Cancer Res. 2020;39(1):284. 33317597 10.1186/s13046-020-01749-x PMC7734811

[11] Kofink D , Muller SA , Patel RS , et al. Routinely measured hematological parameters and prediction of recurrent vascular events in patients with clinically manifest vascular disease. PLoS One. 2018;13(9):e0202682. 30192769 10.1371/journal.pone.0202682 PMC6128486

[12] Bindraban RS , Ten Berg MJ , Haitjema S , et al. Exploring the value of routinely measured hematology parameters for identification of elderly patients at high risk of death at the emergency department. Acute Med. 2018;17(4):188‐202. 30882102

[13] Gijsberts CM , den Ruijter HM , de Kleijn DPV , et al. Hematological parameters improve prediction of mortality and secondary adverse events in coronary angiography patients: a longitudinal cohort study. Medicine (Baltimore). 2015;94(45):e1992. 26559287 10.1097/MD.0000000000001992 PMC4912281

[14] Postow MA , Sidlow R , Hellmann MD . Immune‐related adverse events associated with immune checkpoint blockade. N Engl J Med. 2018;378(2):158‐168. 29320654 10.1056/NEJMra1703481

[15] ten Berg MJ , Huisman A , van den Bemt PM , Schobben AF , Egberts AC , van Solinge WW . Linking laboratory and medication data: new opportunities for pharmacoepidemiological research. Clin Chem Lab Med. 2007;45(1):13‐19. 17243908 10.1515/CCLM.2007.009

[16] Wessels LF , Reinders MJ , Hart AA , et al. A protocol for building and evaluating predictors of disease state based on microarray data. Bioinformatics. 2005;21(19):3755‐3762. 15817694 10.1093/bioinformatics/bti429

[17] LeDell E , Petersen M , van der Laan M . Computationally efficient confidence intervals for cross‐validated area under the ROC curve estimates. Electron J Stat. 2015;9(1):1583‐1607. 26279737 10.1214/15-EJS1035 PMC4533123

[18] Finazzi S , Poole D , Luciani D , Cogo PE , Bertolini G . Calibration belt for quality‐of‐care assessment based on dichotomous outcomes. PLOS One. 2011;6(2):e16110. 21373178 10.1371/journal.pone.0016110 PMC3043050

[19] Vickers AJ , Elkin EB . Decision curve analysis: a novel method for evaluating prediction models. Med Decis Making. 2006;26(6):565‐574. 17099194 10.1177/0272989X06295361 PMC2577036

[20] R Core Team . R: A Language and Environment for Statistical Computing. R Foundation for Statistical Computing; 2014.

[21] Wang DY , Salem J‐E , Cohen JV , et al. Fatal toxic effects associated with immune checkpoint inhibitors: a systematic review and meta‐analysis. JAMA Oncol. 2018;4(12):1721‐1728. 30242316 10.1001/jamaoncol.2018.3923 PMC6440712

[22] Verheijden RJ , May AM , Blank CU , et al. Lower risk of severe checkpoint inhibitor toxicity in more advanced disease. ESMO Open. 2020;5(6):e000945. 33199288 10.1136/esmoopen-2020-000945 PMC7670947

[23] Bissenova S , Ellis D , Mathieu C , Gysemans C . Neutrophils in autoimmunity: when the hero becomes the villain. Clin Exp Immunol. 2022;210(2):128‐140. 36208466 10.1093/cei/uxac093 PMC9750832

[24] Uffen JW , Oomen P , de Regt M , Oosterheert JJ , Kaasjager K . The prognostic value of red blood cell distribution width in patients with suspected infection in the emergency department. BMC Emerg Med. 2019;19(1):76. 31795936 10.1186/s12873-019-0293-7 PMC6889630

[25] van Solinge WW , Ten Berg MJ , Haitjema S . Data‐driven integrated diagnostics: the natural evolution of clinical chemistry? Ned Tijdschr Geneeskd. 2019;163:D3512. 30875155

