# Supplementary material for: Added diagnostic value of routinely measured hematology variables in diagnosing immune checkpoint inhibitor mediated toxicity in the emergency department
Source: Cancer Med. 2023 Apr 19;12(11):12462–9. doi: 10.1002/cam4.5956 (PMC10278460; doi:10.1002/cam4.5956)
Supplement: Supplementary file 1 — Data S1: [file CAM4-12-12462-s001.docx]

## Supplementary Material

### Supplementary Figures


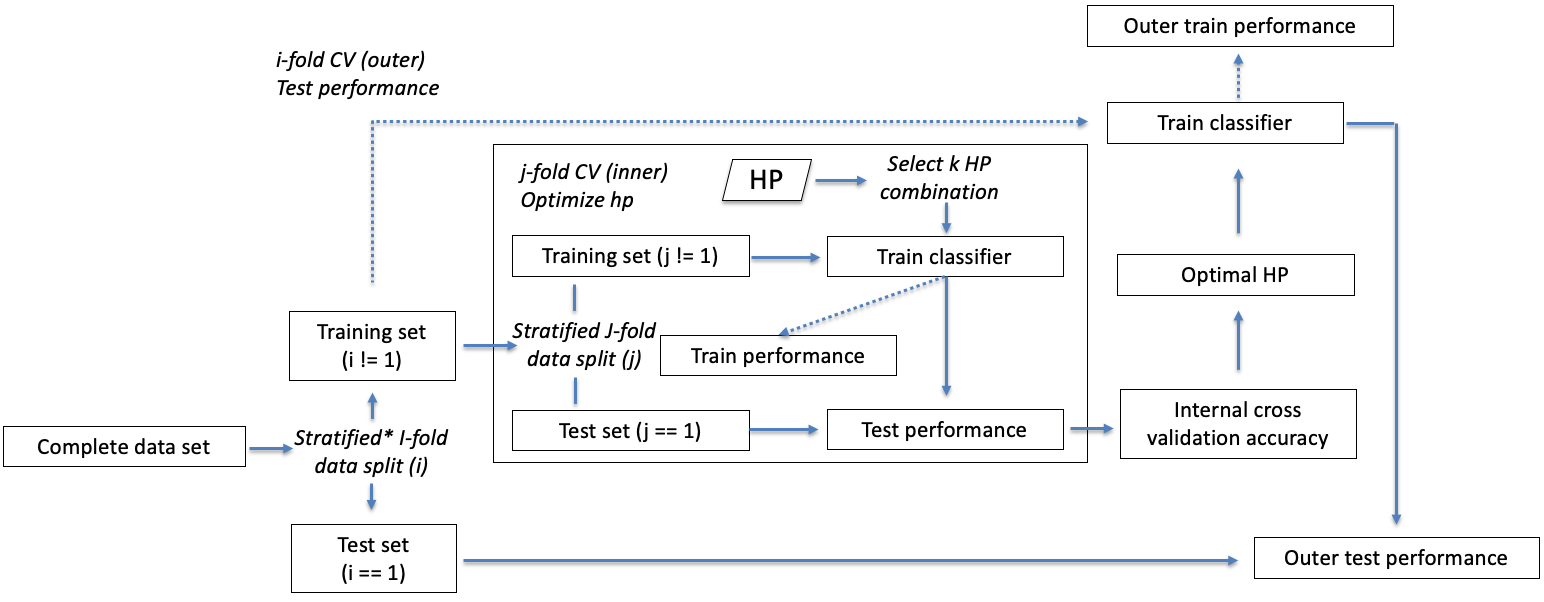


Supplementary Materials Figure 1: scheme of the double loop cross validation (DLCV). HP: hyperparameter, CV: cross validation. *: data was stratified on both label and patient ID.

**Supplemental Materials Figure 2: dendrogram of all 77 haematological variables (*CELL-DYN Sapphire*) computed with Euclidean distance.** Length of each node depicts the similarity between variables. The blue circle represents the 0.80 cut-off. Only one of the clustered variables outside the circle was used in training.

###
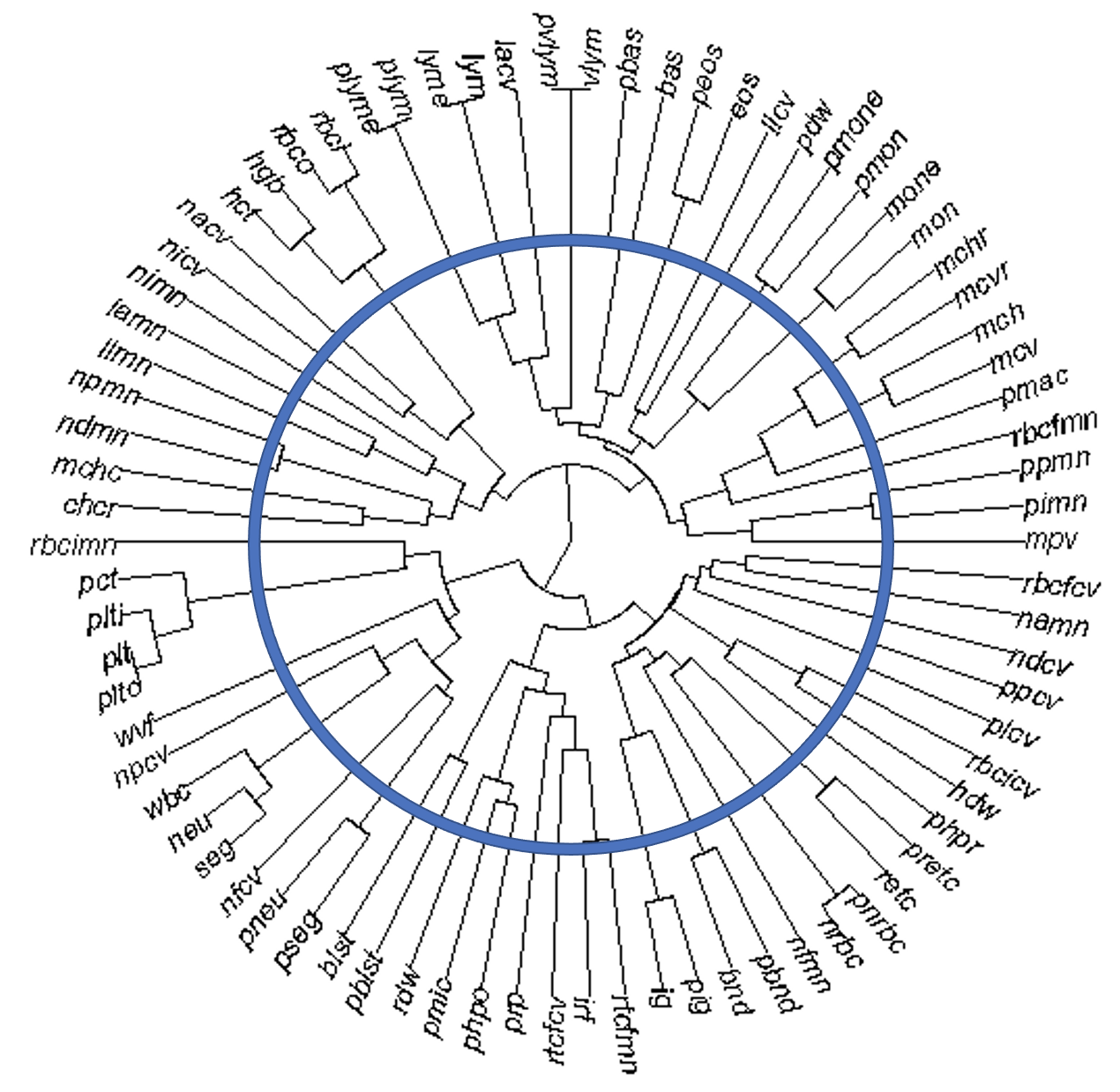


**Supplemental Materials Figure 3: distributions of the four identified variables**

Visits with irAE were graded between 1-5, visits without irAE were assigned with a CTCAE grade of 0.

*
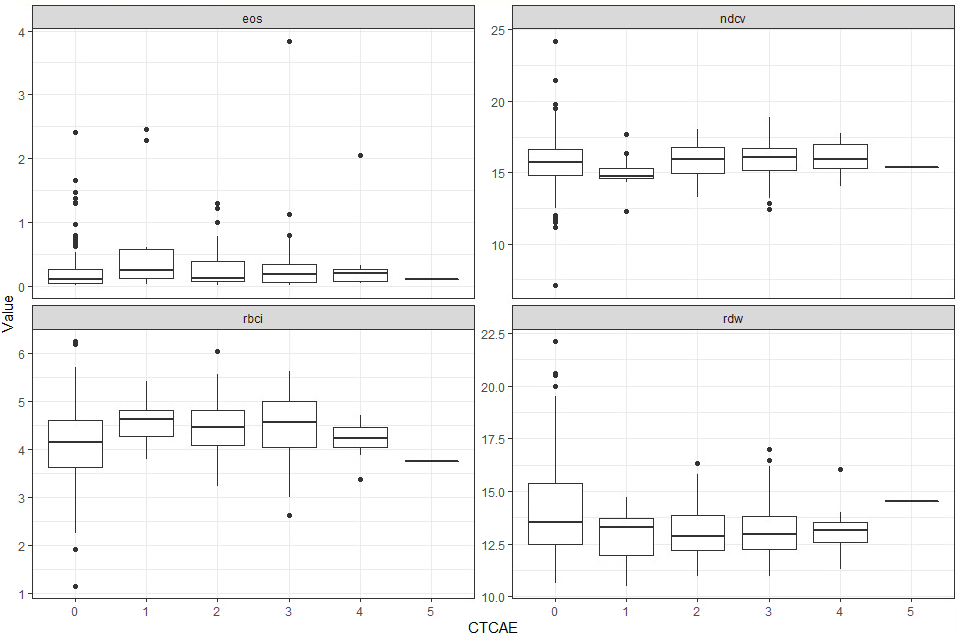
*

### Supplementary Tables

**Supplemental Table 1: total number of diagnoses of immune related adverse events (N = 224), not mutually exclusive.**

| Medical diagnosis | Count (%) |
| --- | --- |
| arthritis  cholangitis  colitis  dermatitis  diabetes  duodenitis  encephalitis  fever (unspecified)  gastritis  hepatitis  hypophysitis  ITP  meningitis  myocarditis  myositis  nephritis  pancreatitis  pericarditis  pleuritic  plexopathy  polymyalgia rheumatica  pneumonitis  radiculitis  sarcoid like reaction  thyroiditis | 2 (0.9%)  2 (0.9%)  61 (27.2%)  15 (6.7%)  4 (1.8%)  2 (0.9%)  5 (2.2%)  1 (0.4%)  4 (1.8%)  29 (12.9%)  24 (10.7%)  1 (0.4%)  7 (3.1%)  1 (0.4%)  2 (0.9%)  4 (1.8%)  2 (0.9%)  1 (0.4%)  1 (0.4%)  1 (0.4%)  2 (0.9%)  40 (17.9%)  1 (0.4%)  3 (1.3%)  9 (4.0%) |

**Supplemental Table 2: description of all 77 *CELL-DYN Sapphire* variables.** Estimated coefficients are shown as mean (±SD). *: discarded variables with less than five unique values. CV: Coefficient of Variance

| Variable | Description |
| --- | --- |
| bas | Basophilic granulocyte absolute count |
| blst | Blast absolute count |
| bnd | Banded granulocyte absolute count |
| chcr | Mean corpuscular HGB concentration per reticulocyte |
| eos | Eosinophil granulocyte absolute count |
| hct | Hematocrit |
| hdw | Hemoglobin distribution width |
| hgb | Hemoglobin USA units |
| ig | Immature granulocyte absolute count |
| irf | Immature reticulocyte fraction |
| lacv | CV of Axial Light Loss |
| lamn | Mean lymphocyte size |
| licv | CV of Intermediate Angle Scattering |
| limn | Intermediate Angle Scattering |
| lym | Lymphocyte absolute count |
| lyme | Lymphocyte (excluding atypical lymphocytes) |
| mch | Mean corpuscular hemoglobin (USA units) |
| mchc | Mean corpuscular hemoglobin concentration (USA units) |
| mchr | Mean corpuscular HGB per reticulocyte |
| mcv | Mean corpuscular volume |
| mcvr | Mean corpuscular volume of reticulocytes |
| mon | Monocyte absolute count |
| mone | Monocytes (excluding blasts) absolute count |
| mpv | Mean platelet volume |
| nacv | Coefficient of variance of neutrophil size |
| namn | Mean neutrophil size |
| ndcv | CV of Depolarized Side Scattering |
| ndmn | Neutrophil Lobularity/Granularity and nuclear lobularity |
| neu | Neutrophilic granulocyte (segments, banded and immature granulocytes) absolute count |
| nfcv | CV of Fluorecent Channel 3 |
| nfmn | Fluorecent Channel 3 |
| nicv | CV of Intermediate Angle Scattering |
| nimn | Intermediate Angle Scattering |
| npcv | CV of Polarized Side Scattering |
| npmn | Polarized Side Scattering |
| nrbc* | Nucleated red blood cells absolute count |
| pbas | Percentage of basophilic granulocytes |
| pblst | Percentage of blasts |
| pbnd | Percentage of banded granulocytes |
| pct | Plateletcrit |
| pdw | Platelet distribution width |
| peos | Percentage of eosinophilic granulocytes |
| phpo | Percent of red blood cells with HGB concentration less than 28 g/dL |
| phpr | percent of red blood cells with HGB concentration more than 41 g/dL |
| picv | CV of Intermediate Angle Scattering |
| pig | Percentage of immature granulocytes |
| pimn | Platelet complexity of intracellular structure |
| plt | Platelet count |
| plti | Platelet count by impedance |
| plto | Platelet count by optics |
| plym | Percentage of lymphocytes |
| plyme | Percentage of lymphocytes (excluding atypical lymphocytes) |
| pmac | Percent of red blood cells with volume greater than 120 fL |
| pmic | Percent of red blood cells with volume less than 60 fL |
| pmon | Percentage of monocytes |
| pmone | Percentage of monocytes (excluding blasts) |
| pneu | Percentage neutrophilic granulocytes |
| pnrbc* | NRBC percentage count per 100 WBC |
| ppcv | CV of Polarized Side Scattering |
| ppmn | Polarized Side Scattering |
| pretc | Percentage of reticulocytes |
| prp | Percentage of reticulated platelets / Enumeration of reticulated platelets |
| pseg | Segmented granulocyte percentage count |
| pvlym* | Percentage of atypical lymphocyte |
| rbcfcv | CV% of FL1 signal |
| rbcfmn | Mean of FL1 signal |
| rbci | Red blood cell count by impedance |
| rbcicv | CV of Intermediate Angle Scattering |
| rbcimn | Intermediate Angle Scattering |
| rbco | Red blood cell count by optics |
| rdw | Red blood cell distribution width |
| retc | Reticulocyte absolute count |
| rtcfcv | CV% of Reticulocyte population during reticulocyte measurement on FL1 signal |
| rtcfmn | Position of Reticulocyte population during reticulocyte measurement on FL1 signal |
| seg | Segmented granulocyte absolute count |
| vlym* | Atypical (variant) lymphocyte absolute count |
| wbc | White blood cell count |
| wvf | White blood cell viability fraction |
